# *Leptospira* surface adhesin (Lsa21) induces Toll like receptor 2 and 4 mediated inflammatory responses in macrophages

**DOI:** 10.1038/srep39530

**Published:** 2016-12-20

**Authors:** Syed M. Faisal, Vivek P. Varma, M. Subathra, Sarwar Azam, Anil K. Sunkara, Mohd Akif, Mirza. S. Baig, Yung-Fu Chang

**Affiliations:** 1 National Institute of Animal Biotechnology, Hyderabad, India; 2 Department of Biochemistry, School of Life Sciences, University of Hyderabad, Hyderabad, India; 3 Centre for Biosciences and Biomedical Engineering, Indian Institute of Technology, Indore, MP, India; 4 Department of Population Medicine and Diagnostic Sciences, College of Veterinary Medicine, Cornell University, Ithaca, NY, USA

## Abstract

Leptospirosis is zoonotic and emerging infectious disease of global importance. Little is understood about *Leptospira* pathogenesis and host immune response. In the present work we have investigated how *Leptospira* modulates the host innate immune response mediated by Toll-like receptors (TLRs) via surface exposed proteins. We screened *Leptospira* outer membrane/surface proteins for their ability to activate/inhibit TLR2/4 signaling in HEK293 cell lines. Of these the 21 kDa *Leptospira* surface adhesin, Lsa21 had strong TLR2 and TLR4 activity leading to production of proinflammatory cytokines and expression of costimulatory molecules in mouse macrophages. This activity of Lsa21 on innate response was dependent on activation of mitogen activated protein kinases (MAPKs) via stimulating the rapid phosphorylation of p38, JNK and activation of transcription factor NF-κB. Additionally, neutralizing antibodies against TLR2 and TLR4 significantly inhibited cytokine secretion and attenuated Lsa21 induced phosphorylation of p38 and JNK. Furthermore, Lsa21 induced cytokine levels were significantly lower in TLR2^−/−^ and TLR4^−/−^ than in wild type mouse macrophage cell lines. Confocal microscopy and molecular docking confirmed that Lsa21 interacted with both TLR2 and TLR4. These results indicate that Lsa21 is a potent TLR2 and TLR4 agonist that induces strong innate response and may play important role in *Leptospira* pathogenesis.

Leptospirosis, a zoonotic and infectious disease, is an emerging global health issue, particularly in warm tropical climates. The disease is caused by the spirochete *Leptospira interrogans,* which is able to survive for years in soil and water and causes fatal infection in farm and domestic animals as well as in humans^1^,^2^. More than 500,000 cases of severe leptospirosis are reported each year, with case fatality rates exceeding 20%^2^. Recently, a much more severe form of the disease called Leptospirosis-Associated Pulmonary Hemorrhage Syndrome (LPHS) has emerged for which the case fatality rate is more than 50%^2^. Antibiotics are effective only during early infection and some are associated with severe reactions^3^,^4^,^5^. Current *Leptospira* vaccines provide limited protection and are unable to prevent the shedding of bacteria in urine of infected animals^6^,^7^,^8^,^9^. Hence there is a need to develop an effective vaccine which requires better understanding of host response against *Leptospira* infection.

Toll-like receptors (TLRs) play important role in restricting the spread of pathogens in the host. Engagement of TLRs by microbial products results in homodimerization and recruitment of the adaptor molecule MyD88 leading to activation of various intracellular signaling pathways such as NF-kB and mitogen-activated protein (MAP) kinases. Activation of these pathways regulate secretion of cytokines, upregulation of costimulatory molecules, resulting in B and T cells activation, proliferation and differentiation, all of which contribute to activation of adaptive immune response thereby allowing the host to eradicate the invading pathogens from the body^10^,^11^,^12^. However, pathogens have evolved countermeasures to evade TLRs and other host mediated immune responses. Recent studies have shown that TLRs specifically TLR2 and TLR4 play critical role in controlling *Leptospira* infection^13^. It is hypothesized that *Leptospira* evades the host immune response by disrupting TLR signaling by varying lipopolysaccharide (LPS) expression or down-regulating expression of surface proteins, thereby establishes infection in various organs^14^,^15^,^16^,^17^,^18^,^19^,^20^,^21^. Several *Leptospira* components (surface proteins and LPS) have been shown to trigger TLR dependent/independent inflammatory reactions during infection^19^,^22^,^23^. LipL32 is a TLR2 agonist and was recently shown to induce early inflammation in human proximal tubule cells and animals^24^. *Leptospira* hemolysins have also been shown to induce proinflammatory response through TLR2 and TLR4^23^.

Based on the previous studies we hypothesized that additional *Leptospira* components (especially surface proteins) may be involved in inducing a strong innate/inflammatory response. We screened several recombinant *Leptospira* surface proteins and observed that recombinant *Leptospira* surface adhesin (rLsa21) induced strong TLR2/4 activity. Lsa21 is a surface exposed adhesin that binds strongly to extra cellular matrix and plays a role in pathogenesis and virulence^25^. Lsa21 also imparted protection against challenge in a hamster model of Leptospirosis^26^. However, the mechanism of how the innate immune system responds to Lsa21 and its downstream signaling pathways remain unclear.

In this study, we used recombinant Lsa21 (rLsa21) and tested its ability to activate innate response through TLRs in mouse macrophage cell lines. We used TLR2^−/−^, TLR4^−/−^ and TLR2^−/−^/4^−/−^ mouse macrophage cell lines stimulated with rLsa21 to test their ability secrete pro-inflammatory cytokines and express costimulatory molecules and surface markers. In addition, we further identified the TLRs and intracellular signaling pathways that are essential for regulating the expression of pro-inflammatory cytokines upon stimulation by rLsa21.

## Results

### Purification and testing of TLR activity of recombinant proteins

**Figure 1:**
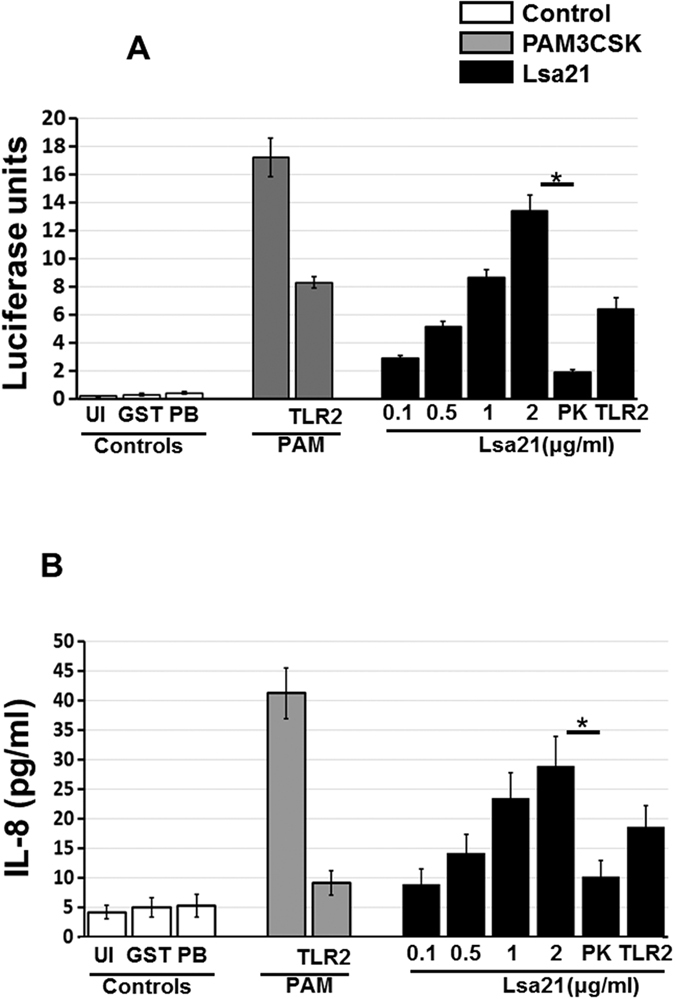
Testing of TLR activity of rLsa21. rLsa21 was screened for TLR2 activity on HEK293 cell lines transfected with TLR2 and NF-κB driven reporter plasmids by Luciferase assay (**A**) and **i**nduction of IL-8 (**B**) after stimulation with the protein and pretreatment with PK or blocking with TLR2 antibodies as described in material and methods. UI indicates uninduced or unstimulated cells, GST- indicates Glutathione S-transferase protein (2 μg/ml) PAM indicates PAM3CSK (20 ng/ml), PK is Proteinase K, PB is Polymixin B (10 μg/ml) and TLR is Toll like receptors.

Various surface proteins of *Leptospira* (LipL32, Lsa21, LigAvar) were expressed as GST fusion protein in soluble form which were purified for further analysis (sup. Fig. 1A). These proteins showed TLR2 activity in dose dependent manner in HEK293 cells transiently expressing TLR2 (sup. Fig. 1B). One of these rLsa21 showed maximum activity which correlated with production of IL-8 (Fig. 1). In particular, polymyxin B treatment did not affect the cytokine inducing ability of rLsa21 whereas the proteinase K plus heating treatment abolished the cytokine-inducing ability of rLsa21. Further, there was significant reduction in cytokine levels when receptors were blocked by TLR2 monoclonal antibody. These results confirm that the observed TLR activity was due to rLsa21 rather than LPS.

### rLsa21 elicits cytokine production from mouse macrophages in a TLR2 and TLR4-dependent manner

**Figure 2:**
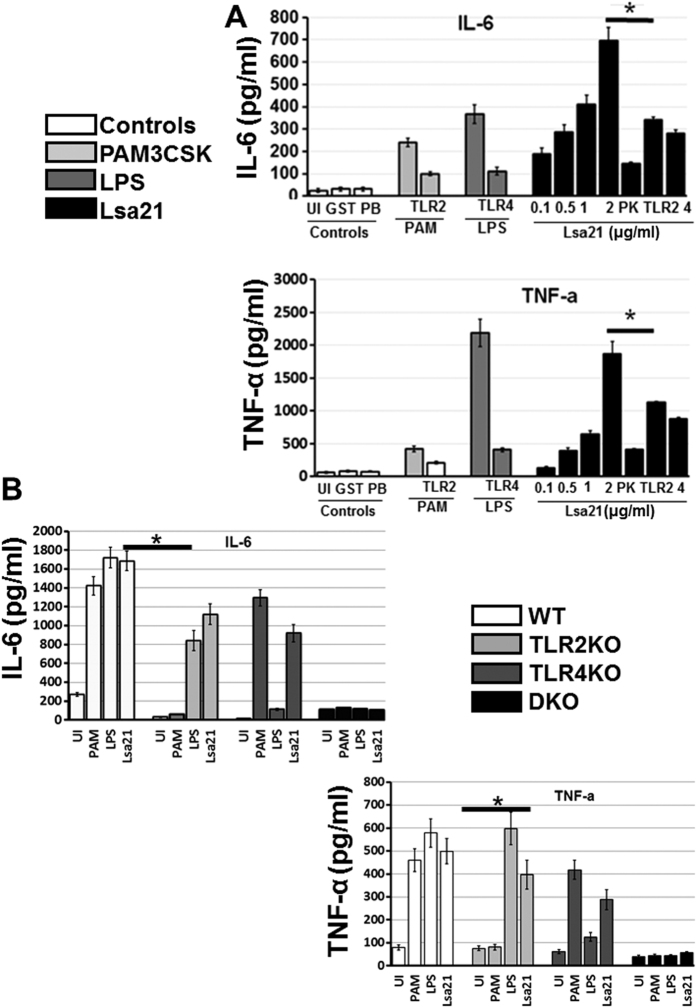
rLsa21 induced cytokine secretion in mouse macrophages. (**A**) RAW264.7 cells were incubated with LPS (500 ng/ml) or PAM3CSK (20 ng/ml) or rLsa21 (0.1, 0.5, 1 or 2 μg/ml) with polymixin B for 24 hrs and supernatant was collected to measure levels of TNF-α and IL-6 by ELISA. rLsa21 (2 μg/ml) pre-treated with PK followed by heating at 95 °C for 15 min, or pre blocking of RAW264.7 cells with TLR2/TLR4 monoclonal Antibodies (Biolegend) was used as controls. (**B**) TLR2^−/−^, TLR4^−/−^ or TLR2^−/−^/TLR4^−/−^ DKO macrophages cell lines were treated with rLsa21 (2 μg/ml), LPS (500 ng/ml) or PAM3CSK (20 ng/ml) and levels of IL-6 and TNF-α in the supernatants were measured with ELISA. Data are representative of three different experiments. Significant differences were calculated using the Student’s t-test (*Indicates P < 0.05). UI indicates uninduced or unstimulated cells, GST- indicates Glutathione S-transferase protein (2 μg/ml) PAM indicates PAM3CSK (20 ng/ml) LPS is *E. coli* Lipopolysaccharide (500 ng/ml), PK is Proteinase K, PB is Polymixin B (10 μg/ml) and TLR is Toll like receptors.

We evaluated TLR activity of rLsa21 in mouse macrophage cell line RAW264.7. Stimulation of RAW264.7 cells with various concentrations of rLsa21, significantly increased the production of TNF-α and IL-6 in a dose-dependent manner (Fig. 2A). The effect was specific to rLsa21, because proteinase K plus heating abrogated the rLsa21 induced production of cytokines. In addition, polymyxin B did not attenuate the rLsa21 induced TNF-α and IL-6 production but inhibited the LPS induced production, indicating that the stimulatory effects observed were not due to contamination with LPS. Further blocking the TLR receptors with monoclonal antibodies against TLR2 and TLR4 also reduced the production of cytokines. Next, we used TLR2^−/−^, TLR4^−/−^ and TLR2^−/−^/4^−/−^ mouse macrophages cell lines to analyze whether TLR2 and TLR4 mediated the production of cytokines induced by rLsa21. Cytokine production was significantly lower in rLsa21- stimulated TLR2^−/−^ and TLR4^−/−^ cell lines as compared to WT and completely abrogated in TLR2^−/−^/4^−/−^ double knockout lines, indicating that the effects of rLsa21 were dependent on TLR2 and TLR4 (Fig. 2B). These results suggest that the rLsa21 induces inflammatory cytokine production in TLR2 and TLR4 dependent manner.

### rLsa21 activates MyD88-dependent and TRIF-dependent signaling pathways

**Figure 3:**
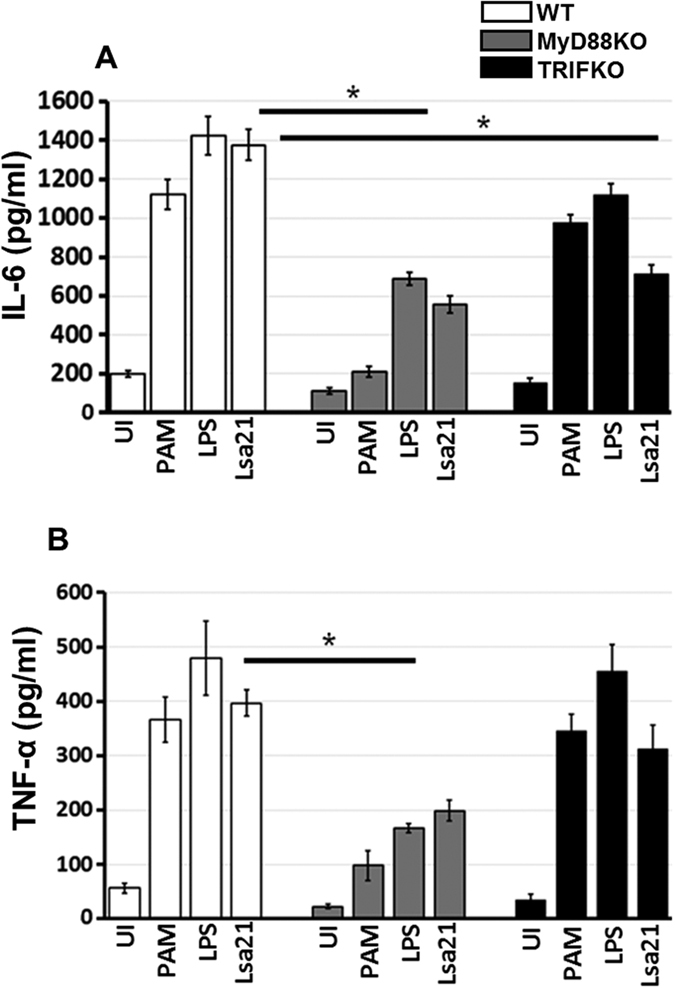
rLsa21 induced cytokine secretion in MyD88^−/−^ and TRIF^−/−^ mouse macrophages. WT, MyD88^−/−^ and TRIF^−/−^ peritoneal macrophages cell lines were treated with rLsa21 (2 μg/ml), LPS (500 ng/ml) or PAM3CSK (20 ng/ml) and levels of IL-6 (**A**) and TNF-α (**B**) in the supernatants were measured with ELISA. Significant differences were calculated between control and treatment groups using the Student’s t-test (*Indicates P < 0.05). UI indicates uninduced or unstimulated cells, PAM indicates PAM3CSK (20 ng/ml) and LPS is *E. coli* Lipopolysaccharide (500 ng/ml).

MyD88 is a major adapter molecule that is downstream of TLR2 and TLR4. Using MyD88^−/−^ mouse macrophage cell lines, we showed rLsa21 induced cytokine production was remarkably reduced (p < 0.05) compared to that rLsa21 induced TNF-α and IL-6 in WT mouse macrophages indicating the use of a MyD88-dependent pathway (Fig. 3A). TLR4 can also recruit another adapter molecule, TRIF, to transduce an activation signal. We used TRIF^−/−^ mouse macrophage cell lines and analyzed production of TNF-α and IL-6 induced by rLsa21. The result showed that rLsa21 induced IL-6 production in TRIF^−/−^ cells was significantly lower than in WT cells (P < 0.05; Fig. 3B), however there was no effect on production of TNF-α indicating that a TRIF-dependent signaling pathway was also involved in production of cytokines by rLsa21.

### rLsa21 is a ligand for mouse TLR2 and TLR4

**Figure 4:**
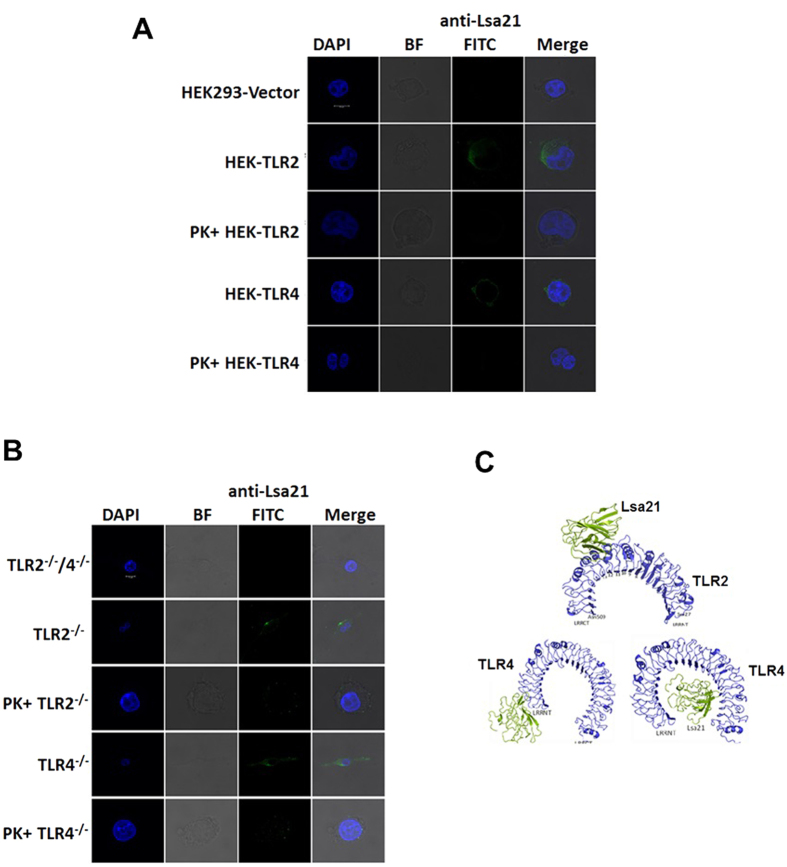
Interaction/binding of rLsa21 with TLR2 and TLR4. (**A**) HEK293-TLR2, HEK293-TLR4 and HEK293-vector cells were cultured in cell imaging dishes and then incubated with rLsa21 (2 μg/ml) or rLsa21 (2 μg/ml) treated with Proteinase K for 30 min. After washing, the cells were fixed with 4% PFA and then sequentially incubated with mouse anti-rLsa21 serum and FITC Goat anti-mouse IgG. After washing, mounting medium was added and then viewed and photographed with confocal fluorescence microscopy. Data are representative of three independent experiments. (**B**) TLR2^−/−^, TLR4^−/−^ or TLR2^−/−^/TLR4^−/−^ DKO macrophages cell lines were incubated with rLsa21 (2 μg/ml) or rLsa21 (2 μg/ml) treated with Proteinase K for 30 min. After washing cells were fixed and stained with respective antibodies as described in materials and methods. (**C**) Molecular modeling of interaction between Lsa21 (green) and LRR domain of TLR2 (blue). All 10 top ranking docking complexes showed that the Lsa21 binding site lies at convex region of TLR2 and its binding region overlaps with ligand binding and TLR1-TLR2 hetro-dimerization region (LRR- 10, 11, 12). Molecular modeling of interaction between Lsa21 (green cartoon) and TLR4 (blue cartoon). Out of 10 top ranking docking complexes, in three docking complexes Lsa21 was observed to bind to concave region and interact with C-terminal and remaining seven complexes it docked on the N-terminal region of TLR4. BF- indicates bright field, PK indicates Proteinase K treatment.

Because rLsa21 could stimulate cytokine secretion via TLR2 and TLR4 we determined whether it interacted with TLRs using confocal microscopy. Immunofluorescence images showed strong anti- rLsa21 fluorescence on the surface of HEK293-TLR2 and HEK293-TLR4 cells incubated with rLsa21 but very little or no fluorescence on HEK293-vector control cells (Fig. 4A). Further there was reduced fluorescence in cells (HEK293-TLR2 and HEK293-TLR4) incubated with Proteinase K treated Lsa21 (Fig. 4A). These results indicated that rLsa21 interacts specifically and primarily with TLR2 and TLR4. Similar results were obtained with TLR^−/−^ cell lines where strong anti- rLsa21 fluorescence was observed on the surface of TLR2^−/−^ and TLR4^−/−^ cells incubated with rLsa21 but little fluorescence on TLR2^−/−^/4^−/−^ cells (Fig. 4B). Further there was reduced fluorescence in cells (TLR2^−/−^ and TLR4^−/−^ cells) incubated with Proteinase K treated Lsa21. Molecular docking models confirmed the binding of Lsa21 with TLR2 and TLR4. We used a 3D-dock (http://www.bmm.icnet.uk/docking) molecular docking program using shape complementarity to assess the interaction of protein molecules, to predict possible sites for interaction between TLR2 and rLsa21. The top ranking docking scores for rLsa21 was found to be located at the convex region that stretches the border of the central and C-terminal domain (overlapping with the LRR 12–15) of TLR2 (Fig. 4C). However, either the concave or C terminal region of TLR4 receptor was predicted to be the Lsa21 binding site. These data demonstrated that rLsa21 stimulates cytokine secretion specifically via binding to TLR2 and TLR4.

### rLsa21-induced cytokine production depends primarily on the activation of p38, JNK and NF-κB dependent signaling

**Figure 5:**
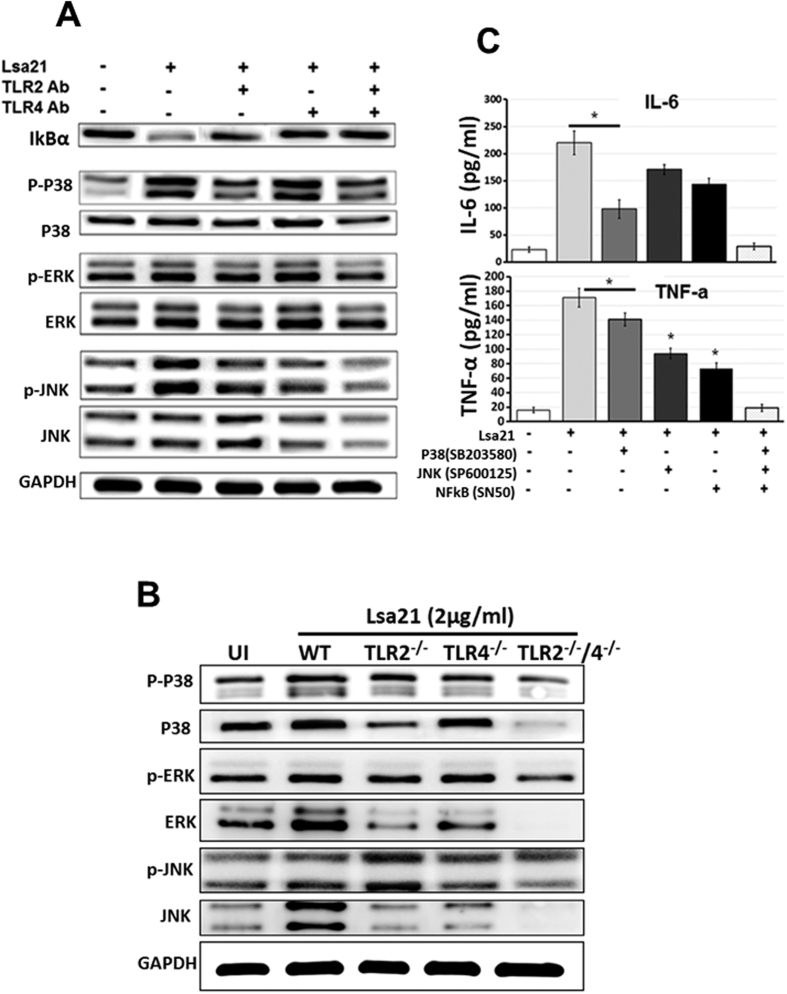
Analysis of signaling pathways involved in rLsa21-mediated cytokine production in mouse macrophages. (**A**) RAW264.7 cells were pretreated for 30 min at 37 °C with anti-TLR2 (30 μg/ml), anti-TLR4 (30 μg/ml), or an isotype control Ab (30 μg/ml) before stimulation with rLsa21 (5 μg/ml) for 30 min. Levels of phosphorylated p38, JNK, and ERK1/2 induced by rLsa21 and degraded IκBα were analyzed by western blot using anti–phospho-p38 (p-p38), or anti–phospho-ERK1/2 (p-ERK1/2), anti–phospho-JNK (p-JNK) and anti-mouse IκBα as well as a specific control Ab for each of the unphosphorylated kinases. Data are representative of those obtained in three independent experiments. (**B**) TLR2^−/−^, TLR4^−/−^ or TLR2^−/−^/TLR4^−/−^ DKO macrophages cell lines were stimulated with rLsa21 (5 μg/ml) for 30 min. Levels of phosphorylated p38, JNK, and ERK1/2 induced by rLsa21 were analyzed by western blot as described in materials and methods. (**C**) RAW 264.7 cells were pretreated for 30 min with NF-kB inhibitor (SN50; 20 μM), JNK inhibitor (SP600125; 40 μM) or p38MAPK inhibitor (SB203580; 30 μM) and then stimulated with rLsa21 (2 μg/ml). After incubation, supernatants were harvested and levels of IL-6 and TNF-α were measured by ELISA. Data are representative of three different experiments. (*Indicates P < 0.05).

Because MAPKs are critical factors that are involved in cellular responses to inflammatory stimuli, we examined the activation of MAPKs in response to rLsa21. Western blotting was used to examine the phosphorylation of p38, JNK, ERK1/2 and IkBα in RAW264.7 cells at different time points after stimulation with rLsa21 (2 μg/ml). Strong phosphorylation of p38, JNK and ERK1/2 was observed after 10–60 min of stimulation. Little phosphorylation was observed in untreated cells. Maximum phosphorylation was observed after 30 min of stimulation with rLsa21 (data not shown). We used this time point to determine phosphorylation after blocking with TLR antibody. Blocking with TLR2 and TLR4 antibody significantly reduced the phosphorylation of p38 and JNK and also inhibited degradation of IkBα (Fig. 5A). Similar results were obtained with TLR2^−/−^, TLR4^−/−^ and TLR2^−/−^/4^−/−^ DKO cell lines stimulated with rLsa21. The rLsa21 mediated phosphorylation of p38 and JNK was significantly reduced in TLR2^−/−^ and TLR4^−/−^ cell lines after 15 min (Fig. 5B). Therefore, the phosphorylation of MAPKs in response to rLsa21 was dependent on both TLR2 and TLR4 binding. To determine which pathway is required for rLsa21 induced cytokine production, different inhibitors of these pathways were tested. ELISA was used to examine rLsa21 induced production of TNF-α and IL-6 in RAW264.7 cells pre-treated with or without NF-kB inhibitor (SN50; 20 μM), JNK inhibitor (SP600125; 40 μM) or p38MAPK inhibitor (SB203580; 30 μM). IL-6 production was significantly blocked by p38 inhibitor (P < 0.05, 50% inhibition with 2 μg/ml rLsa21) and partially by JNK and NF-kB SB203580 (P < 0.05, 30% inhibition with 2 μg/ml rLsa21). The production of TNF-α was also significantly blocked by JNK and NF-κB inhibitor (P < 0.05, 60% inhibition). Combination of all three inhibitors completely inhibited rLsa21 induced production of these cytokines (Fig. 5C). All these results suggest that the rLsa21 stimulates the production of proinflammatory cytokines through p38, JNK and NF-kB pathways.

### rLsa21 enhances expression of costimulatory molecules and maturation marker in mouse macrophage cell lines in a TLR2- and TLR4-mediated manner

**Figure 6:**
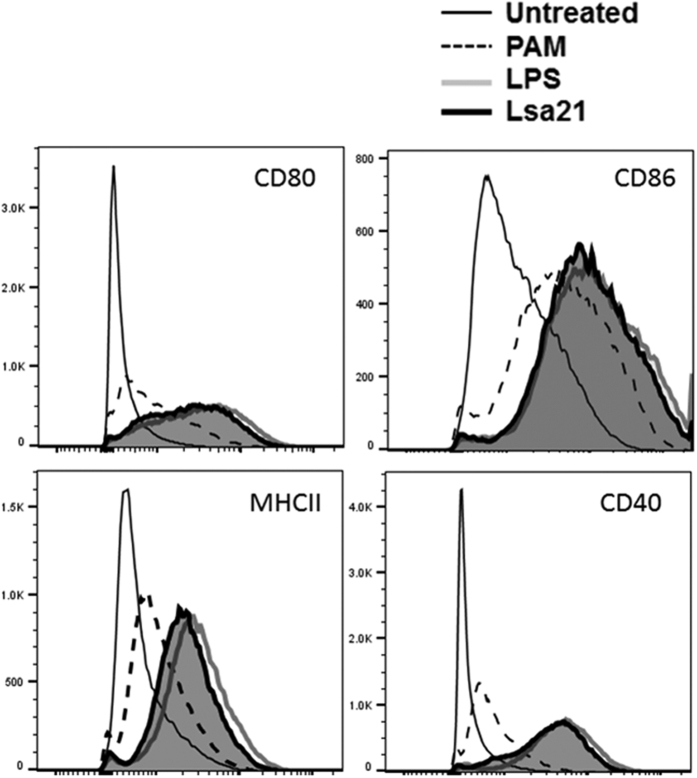
Analysis of rLsa21 induced expression of surface markers on the mouse macrophages. RAW264.7 cells were incubated with LPS (500 ng/ml) or PAM3CSK (20 ng/ml) or rLsa21 (2 μg/ml) for 24 hrs at 37 °C/5% CO_2_ Cells were stained with isotype control Ab or PE-CY5-conjugated anti-MHC-II. FITC-conjugated anti-CD86 or R-phycoerythrin-conjugated anti-CD80 as described in materials and methods and then analyzed by Flow cytometry. UI indicates uninduced or unstimulated cells, PAM indicates PAM3CSK (20 ng/ml) and LPS is *E. coli* Lipopolysaccharide (500 ng/ml).

To investigate the effect of rLsa21 on expression of costimulatory molecules (CD80, CD86, CD40) and maturation marker (MHC-II) which are important for the function of macrophages as antigen presenting cells and phagocytes., RAW264.7 cells were stimulated with rLsa21 for 24 h and the expression of these molecules was analysed using flow cytometry. rLsa21 enhanced the expression of CD80, CD86, MHCII and CD40 in RAW264.7 cells similar to levels observed in LPS stimulated cells (Fig. 6). Hence, in addition to stimulating the production of proinflammatory cytokines, rLsa21 also enhances the expression of surface markers indicating the modulation in antigen presenting activity of the macrophages.

### rLsa21 enhances expression of immune related genes in mouse macrophages in a TLR2- and TLR4-mediated manner

**Figure 7:**
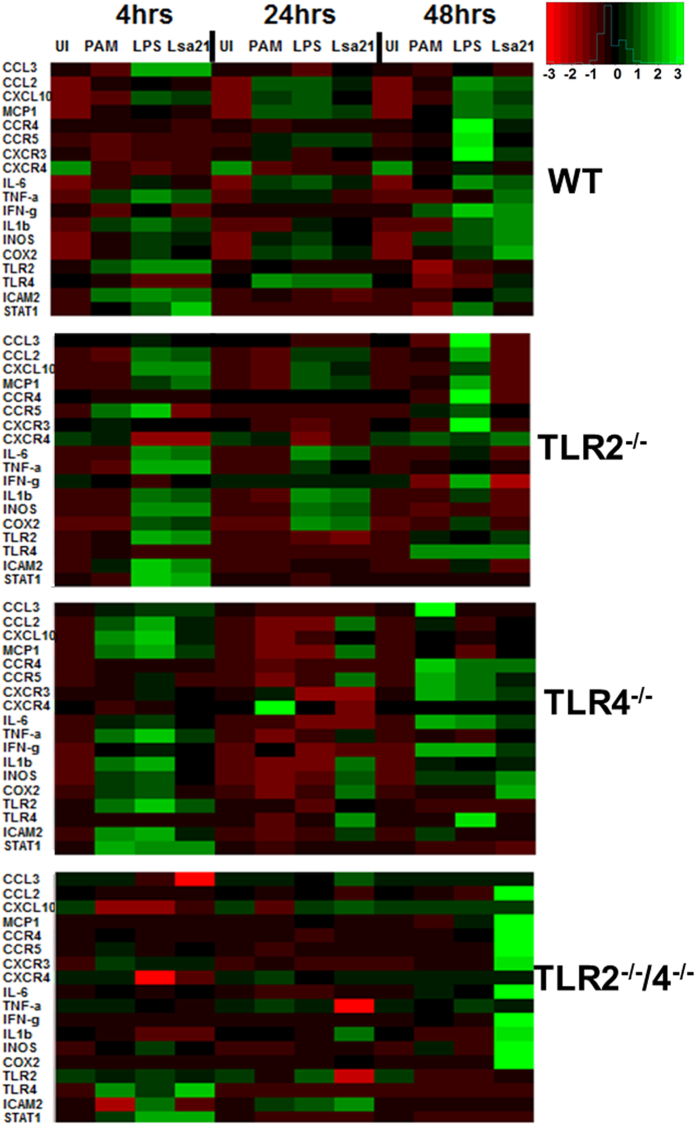
rLsa21 induced gene expression profiling in mouse macrophages. WT, TLR2^−/−^, TLR4^−/−^ and TLR2^−/−^/4^−/−^ mouse macrophage cell lines were treated with rLsa21 (2 μg/ml), LPS (500 ng/ml) or Pam3CSK4 (20 ng/ml). After treatment at various time points (4 hrs, 24 hrs, 48 hrs) cells were recovered in 500 μl of TRIzol, RNA was purified and converted to cDNA. RT-PCR was performed in 96 well microtiter plates on an ABI 7500 sequence detection system as described in material and methods. The experimental data were presented as fold changes of gene expression of stimulated cells at various time points relative to control. mRNA levels of the analyzed genes were normalized to the amount of β-actin present in each sample. UI indicates uninduced or unstimulated cells, PAM indicates PAM3CSK (20 ng/ml) and LPS is *E. coli* Lipopolysaccharide (500 ng/ml).

Expression levels of key inflammatory cytokines/chemokines genes at various time points were determined in WT, TLR2^−/−^, TLR4^−/−^ and TLR2^−/−^/4^−/−^ mouse macrophage cell lines in response to Lsa21. Expression levels of CCL2, CCL10, COX2, IL-1β, IL-6, TNF-α, MCP-1, IFN-γ and iNOS were significantly upregulated in WT mouse macrophage cell lines stimulated with Lsa21 (Fig. 7). The expression of these genes were significantly reduced or down regulated in TLR2^−/−^ and TLR4^−/−^ cell stimulated with Lsa21. However in TLR2^−/−^/4^−/−^ stimulated cells few of these genes were changed. PAM3CSK and LPS showed significant upregulation of genes involved in TLR2 and TLR4 signaling as expected.

## Discussion

*Leptospira* causes persistent infections in humans by evading the TLR mediated host immune response through multiple mechanisms such as limiting the expression or antigenic variation of its membrane proteins, their access to antibodies and modifying its LPS^14^,^16^,^27^. Outer membrane/surface proteins play an important role in the virulence of *Leptospira*^28^. Many of these proteins are pro-inflammatory as they can activate innate immune cells such as macrophages and dendritic cells (DCs) and also enable the *Leptospira* evade the immune system and adhere to the host^29^,^30^,^31^,^32^,^33^. Some of these proteins have also shown promise in development of subunit vaccines^34^,^35^,^36^,^37^. Thus, an understanding of how surface proteins interact with the host immune system and elucidating the molecular mechanisms of this immunomodulation will lead to a greater understanding of the inflammatory processes, innate and adaptive immune responses associated with leptospirosis that may contribute to vaccine development.

Recent studies have identified some of the surface proteins of *Leptospira* that bind to pattern recognition receptors such as toll-like receptors (TLR2,4) and modulate the innate immune response^19^,^22^,^23^. However, most of the surface proteins have not been well-studied with respect to the mechanism of activation of TLR mediated immune response. Here we have shown that rLsa21 is potent agonist of TLR2 and TLR4. We demonstrate that, indeed, rLsa21 induced production of pro-inflammatory cytokines through a TLR2- and TLR4-dependent pathway, because the production of those cytokines was lower in macrophages blocked with TLR2 and TLR4 antibodies or from TLR2^−/−^, TLR4^−/−^ and TLR2^−/−^/4^−/−^ macrophage cell lines (Fig. 2). In addition, rLsa21 maintained stimulating activity when co-cultured with polymyxin B and lost its activity after digestion with proteinase K. These results indicated that the stimulating effect was not due to LPS contamination but rather a specific property of the rLsa21 protein. Although most lipoproteins signal via TLR2, but our data showed that rLsa21 signals via both TLR2 and TLR4. This finding is consistent with various previous reports demonstrating the production of proinflammatory cytokines and chemokines through TLR2/4 by *Leptospira* hemolysins^23^, whole *Leptospira* or their purified components (LPS, membrane proteins) but also proteins from other bacterial pathogens like *Brucella* and *Mycobacterium*^38^,^39^,^40^,^41^. Our results showed that rLsa21 induced cytokine production mainly through MYD88 dependent signaling pathway as there was a reduced concentration of cytokines (IL-6 and TNF-α) in MyD88^−/−^ macrophage cell lines (Fig. 3A). However, reduced production of IL-6 was observed in Lsa21 stimulated TRIF^−/−^ cells, although there was no effect on TNF-α production indicating that Lsa21 is also signaling through TRIF pathway (Fig. 3B). It is not surprising that rLsa21 is signaling through TRIF as TLR4 receptor is also involved and it has recently been shown that adapter molecule TRIF contribute to defense against *Leptospira*^42^. Pathogenic *Leptopspira* induces strong TLR/MYD88 independent inflammatory response hence it is likely that Lsa21 is downregulated and there is involvement of other membrane components in TLR/MYD88 independent inflammatory response.

rLsa21 stimulated cytokine production via binding both TLR2 and TLR4 as strong fluorescence signals were obtained in HEK-TLR2 and HEK-TLR4 cells incubated with Lsa21 which was reduced when the protein was treated with Proteinase K and there was very little or no fluorescence signal in HEK-Vector (Fig. 4A). This was further confirmed in TLR2^−/−^, TLR4^−/−^ cells (Fig. 4B). Molecular docking studies also showed interaction of Lsa21 with both TLR2 and TLR4; binding to TLR2 occurs at convex face on LRR 12–15 whereas TLR4 binding occurs on either C terminal or concave side (Fig. 4C). The Lsa21 binding models complement confocal microscopy experiments showing rLsa21-TLRKO cell binding (Supp Fig. 2). The anti-TLR2 antibody was not able to bind to TLR4^−/−^ (no fluorescence was observed) and is likely to be sharing the same binding region as Lsa21, while strong binding was shown by the anti-TLR4 antibody in TLR2^−/−^ cells probably due to differences in the binding site of Lsa21 (Supp Fig. 2). Pam3CSK4, a synthetic TLR2 ligand is known to interact in a similar way with TLR2 through a different region; thus it is likely that binding of ligands to different site on TLR receptors may lead to conformational changes and trigger different downstream signaling^43^. Further studies will be required to identify the motif (region in rLsa21) which binds to TLRs. To this aim, current efforts are directed at truncating/mutating Lsa21 to determine the possible binding sites.

It is known that interaction of a ligand with TLR2 or TLR4 activates cytokine encoding gene transcription through NF-kB, JNK or p38 signaling pathways^44^. To identify the signaling mechanism/pathways involved in production of proinflammatory cytokines by Lsa21 we used monoclonal antibodies blocking TLR2 and TLR4 receptors on RAW cells and also pharmacological inhibitors of MAP kinases (p38, JNK) and the NF-kB signaling pathway. Our results showed that upon stimulation with rLsa21, p38, ERK and JNK were rapidly phosphorylated and phosphorylation was significantly reduced when receptors were blocked with TLR2 and TLR4 monoclonal antibodies. (Fig. 5A) The degradation of IkBα in our studies suggests translocation of NF-κB to nucleus and provides indirect evidence of involvement of an NF-kB signaling pathway. Similar results were obtained with stimulation of TLR2^−/−^, TLR4^−/−^ and TLR2^−/−^/4^−/−^ macrophage cell lines with Lsa21 (Fig. 5B). The double bands observed in western blot indicate that both the splicing forms/isoforms of JNK (JNKα-54kDa and JNKβ-46kDa) and ERK (ERK1–44kDa and ERK2–42kDa) were phosphorylated by Lsa21. p38 has four isoforms (p38α, p38β, p38γ and p38δ) however we used antibody against p38α which best characterized and is expressed in most cell types. However, phosphorylated p38 (p-p38) has two isoform and both were phosphorylated upon stimulation with Lsa21. Moreover, the inhibitors (SN50, SP600125 and SB203580) drastically reduced the Lsa21 induced production of proinflammatory cytokines (Fig. 5C). These results indicate that p38, JNK and NF-kB are critical in production of cytokines by Lsa21 stimulated macrophages where p38 is involved in production of IL-6 and JNK and NF-κB is involved in production of TNF-α (Fig. 5C). It is important to note that different PAMPs from various pathogens may activate different signaling pathways for production of proinflammatory cytokines. For instance, JNK and NF-κB signaling pathway is activated by *Leptospira* hemolysins^23^, p38 and JNK is activated by BCSP31 of *Brucella*^40^ and MPT83 of *M. tuberculosis*^39^, NF-κB by streptococcal hemolysins^45^ and p38 and NF-κB by streptolysin O and Listeriolysin^46^,^47^.

The ability of activated macrophages to enhance adaptive immune response depends on expression of costimulatory molecules (CD80, CD86, CD40) and maturation marker (MHCII). Our data shows that Lsa21 significantly enhanced the expression of these molecules on RAW264.7 cells in TLR2/4 dependent manner (Fig. 6). This is in agreement with several previous studies showing enhancement of these molecules by surface proteins from other bacterial pathogens^39^,^40^. Further, the enhanced expression of MHCII by rLsa21 indicates activation of NF-κB signaling. It has been observed that inhibition of NF-kB signaling in DCs decreases expression of MHCII and costimulatory molecules, thereby decreasing the priming of T cells^48^. However, whether the activated macrophages will be able to activate Lsa21 specific adaptive immune response needs to be tested.

TLR activation by microbial surface components (proteins/LPS) leads to transcriptional upregulation of number of genes including cytokines, chemokines and adhesion molecules that are involved in generation of environment where infection can be contained and subsequently cleared^10^,^49^,^50^,^51^. Our RT-PCR analysis also showed that Lsa21 induced upregulation of genes involved in TLR signaling and innate immunity which was significantly reduced or downregulated in cells lacking TLR receptors (TLR2^−/−^, TLR4^−/−^ and TLR2^−/−^/4^−/−^ cells). Most prominent genes upregulated were of IL-6, TNF-α, IL-1β, IFN-γ, MCP-1 and iNOS (Fig. 7). iNOS and IFN-γ are two important mediators of bacterial killing. Previous studies have shown that *Leptospira* OMPs and LipL32 stimulate expression of CCL2/MCP-1 in association with increased generation of iNOS and TNF-α^19^,^20^. Some cytokine/chemokines induced by Lsa21 in TLR2^−/−^/4^−/−^ cells seems not be signaled through TLR2 and TLR4 suggesting that other receptors might be involved in recognition of this ligand (Lsa21). The formation of heterodimers (TLR1-TLR2, TLR2-TLR6) which dictates specificity of ligand recognition may also contribute to difference in cytokine profile with different ligands like PAM3CSK or LPS^52^.

*Leptospira* present wide range of surface molecules (proteins) to the host immune system that may initiate a destructive immune response leading to damage of various vital organs as it establishes persistent infection. Hence, elucidating the mechanism by which *Leptospira* components are detected by TLRs leading to subsequent inflammation as presented in current study seems crucial to achieve a better understanding of the events leading to leptospirosis which will enable the development of effective vaccines. In conclusion, our study has demonstrated that *Leptospira* surface adhesin (Lsa21) induces pro-inflammatory cytokine production via signaling through TLR2 and TLR4.

## Materials and Methods

### Animals, cell lines and reagents

C57BL6 mice were purchased from National Institute of Nutrition and maintained in a pathogen-free condition at NIAB animal facility. The animal care and all the experimental procedures were performed in accordance with the guidelines approved by Institutional Animal Ethics Committee (IAEC) of National Institute of Animal Biotechnology (NIAB). A mouse macrophage cell line (RAW264.7) human embryonic kidney cell line (HEK293) were originally purchased from the American Type Culture Collection (Manassas, VA). WT (NR- 9456), TLR2^−/−^(NR-9457), TLR4^−/−^(NR-9458), TLR2^−/−^/4^−/−^ DKO (NR-19975) cell lines were obtained from BEI Resources, USA. Cells were cultured in DMEM (Sigma, USA) supplemented with 10% FBS (Invitrogen, Carlsbad, CA, USA), penicillin (100 U/ml), and streptomycin (100 mg/ml) and maintained at 37 °C in a humidified incubator (5% CO_2_). Anti-TLR2 and anti-TLR4 were purchased from Biolegend, USA and the inhibitors NF-kB (SN50), p38 (SB203580) and JNK (SP600125) were from Invivogen and Sigma-Aldrich (St. Louis, MO). Human and mouse IL-8, IL-6 and TNF-α sandwich ELISA kits were from R&D biosystems. PerCP-CY5.5–conjugated anti MHC-II, PE-conjugated CD80, APC conjugated CD86 and BV421- CD40 for flow cytometry experiments were procured from BD biosciences, US. All reagents were purchased from Sigma unless otherwise specified.

### Expression and purification of recombinant proteins

The plasmid pGEX4T2 harboring genes encoding Lsa21, LipL32 and variable region of LigA (LigAvar) were constructed previously and subsequently expressed as GST fusion proteins^34^,^36^. The plasmid constructs were transformed into BL21 DE3 and resulting transformants were grown at 37 °C overnight in 1 L of LB broth containing ampicillin (100 μg/ml); the expression of the protein was induced with 1 mM isopropyl β-D-1-thiogalactoside (IPTG). The cells were harvested by centrifugation at 10,000 rpm and the cell pellet was resuspended in 100 mM Tris HCl, 150 mM Nacl pH8.0 and followed by sonication at constant pulses. The lysate was centrifuged to remove cell debris and the supernatant was subjected to affinity chromatography using Glutathione Sepharose 4B column (Thermo Fischer) columns. The recombinant GST fusion proteins were eluted in 100 mM Tris-Hcl, 150 mM Nacl, 10 mM reduced glutathione pH 8.0 according to the manufacturer’s instructions (Thermo Fischer). The GST was cut with thrombin (20 U/ml in PBS, pH 7.3) while the fusion protein was bound to the column by incubating at 4 °C for 12 to 16 h. The thrombin was removed by passing through a HiTrap Benzamidine FF Affinity Column (Amershem Biosciences) following the manufacturer’s protocol. The proteins were eluted and checked for size and purity by SDS-PAGE. The protein was then passed through Detoxi- Gel (Pierce, USA) to remove any contaminating LPS from *E. coli* and residual trace amount of LPS was monitored by Limulus ameobocyte lysate (LAL, Endotoxin Detection Kit, MP Biomedicals, USA) assay following the manufacturer’s instructions. The concentration of purified protein was estimated using Bradford reagent (Thermo Fischer).

### Luciferase assay

HEK293 cells were cultured in DMEM medium and transfected with TLR2, TLR4 and NF-kB reporter plasmids using lipofectamine 3000 following manufacturer’s protocol. Cells were stimulated with the recombinant proteins (10 μg/ml) for 24 hrs and then supernatant was collected for analysis of IL-8 by cytokine ELISA kit (R&D). Cells were lysed using reporter lysis buffer and luciferase activity was determined using a dual luciferase assay kit (Promega) following manufacturer’s protocol. To rule out the possibility of LPS effect, before stimulating with proteins, the preparation was heat denatured at 100 °C for 30 min or treated with proteinase K (1/20 concentration of protein) for 1hr; proteinase K was inactivated by boiling. The protein was added with polymyxin B (10 μg/ml) for 30 min before adding to cell culture to neutralize the effect of any contaminating LPS.

### Cytokine ELISAs

Cytokine ELISA kits (R&D systems) were used to measure cytokine levels, following the manufacturer’s instructions. In experiments designed to block TLR signaling, RAW264.7cells were pretreated for 30 min at 37 °C with a mouse Ab against TLR2 (30 μg/ml) or TLR4 (30 μg/ml). In separate experiment Wildtype, TLR2^−/−^, TLR4^−/−^ and TLR2^−/−^/4^−/−^ cell lines were stimulated with PAM3CSK4 (20 ng/ml), LPS (500 ng/ml) and Lsa21 (2 μg/ml) for 24 hrs at 37 °C and cytokines (IL-6, TNF-α) were measured in culture supernatant by ELISA kit. To access the signaling pathway, additional experiments were performed in which RAW264.7 cells were pretreated for 30 min at 37 °C with inhibitors to NF-kB inhibitor (SN50; 20 μM), JNK inhibitor (SP600125; 40 μM) or p38MAPK inhibitor (SB203580; 30 μM). The RAW264.7cells were then incubated with rLsa21 for 24 h at 37 °C and cytokines were measured.

### Flow cytometric analysis

Cells were incubated in 24-well plates (10^6^ cells/well) with PAM3CSK4 (20 ng/ml), LPS (500 ng/ml) or rLsa21 (2 μg/ml) for 24 h. Cells were harvested and washed with pre-chilled PBS and then blocked for Fc receptors using Fc block (1 μg) for 30 min on ice. They were then incubated on ice for 1 h in the dark with PerCP-CY5.5–conjugated anti MHC-II, PE-conjugated CD80, APC conjugated CD86 and BV421- CD40 (BD biosciences, USA). The cells were washed and fixed with 1% paraformaldehyde. A total of 50,000 events were acquired using a BD FACS Fortessa and the data represents positive staining with specific antibody in reference to respective isotype controls. All the data were analyzed by using FlowJo software (Tree Star Inc.).

### Preparation of Antisera

Female C57 BL6 mice were immunized subcutaneously on days 0 with 20 μg of GST cut Lsa21 in complete Freund’s adjuvant (CFA) and then boosted on day 21 and 28 with 10 μg of Lsa21 in Incomplete Freund’s adjuvant (IFA). Sera was collected one week after last immunization and titer was determined using ELISA. The mouse serum having Lsa21 antibody was used in confocal microscopy.

### Protein-Protein docking studies

Amino acid sequence of Lsa21 (from residues 19–164, signal sequence deleted) was submitted to blastp to find a structural homologue as a template used for homology modeling. However, no significant structural homology was observed. Hence, the amino acid sequence of Lsa21 was submitted for 3-dimensional structural prediction using I-TASSER server^53^ which generated five top best predicted structures with high C-score. A structure in agreement with Ramachandran plot was selected for further energy minimization using steepest descent algorithm in NOMAD-REF online program (http://lorentz.dynstr.pasteur.fr/docking/submission.php)^54^. The energy minimized structure was used to create a docking model on human TLR2 and TLR4 structure using ZDOCK server (http://zdock.umassmed.edu)^55^. The atomic coordinates of TLR2 were taken from the crystal structure of TLR1-TLR2 heterodimer in complex with tri-acylated lipopeptide (2Z7X)^56^, while those ofof TLR4 were taken from crystal structure of TLR4-MAD-2 complex (3FXI)^57^ as static structures (receptor) for docking whereas atomic coordinate of minimized Lsa21 modeled structure was kept as mobile. The docking program generated top 10 scoring complexes based on shape complementarity, electrostatics and rigid body.

### TLR binding assay

An HEK293-TLR2, HEK293-TLR4 and HEK293-Vector stable cell line or TLR2^−/−^, TLR4^−/−^ and TLR2^−/−^/4^−/−^ DKO cell lines were grown overnight on cell imaging dishes (Eppendorf) having glass bottom and then incubated for 30 min at 37 °C with rLsa21 (2 μg/ml) in DMEM without FBS. The cells were washed with PBS and fixed for 15 min using 4% paraformaldehyde. Then the cells were stained by sequential 1-h incubations with 1:100 dilution of mouse anti-Lsa21 (mouse serum) and FITC or Alexa Flour 647 conjugated rabbit anti-mouse IgG (Biolegend, USA), PE-conjugated rabbit anti-mouse TLR2 and APC conjugated rabbit anti-mouse TLR4. Between each staining step, the cells were washed three times with 1% BSA in PBS. After staining, the cells were extensively washed and mounted with VECTA SHIELD with DAPI mounting medium (Biolegend) and observed using a 63X oil objective on Confocal microscope (Leica).

### Western blot analysis

RAW264.7cells were stimulated with rLsa21 and cells were recovered at various time points and treated with RIPA cell lysis buffer supplemented with proteinase and phosphatase inhibitor mixture (Roche Molecular Biochemicals, Indianapolis, IN). The cells were centrifuged at 4 °C, 12,000 × g, for 30 min, and protein concentrations were measured using the BCA protein-quantification assay. Equal amounts of protein were separated by SDS-PAGE and then transferred electrophoretically to PVDF membranes (Millipore, Bedford, MA). After blocking with 5% nonfat milk in PBS (pH 7.4, 0.05% (v/v) Tween-20), the membranes were incubated overnight at 4 °C with primary Abs, including rabbit anti-mouse-p38, anti–mouse phospho-p38, anti-mouse-ERK2, anti–mouse phospho-ERK1/2, anti–mouse stress-activated protein kinase/JNK, anti–mouse phospho–stress-activated protein kinase/JNK, anti-mouse IκBα (Cell Signaling Technology, Beverly, MA), according to the manufacturer’s instructions. After washing with PBS, the membranes were incubated for 1–2 h at 37 °C with the appropriate HRP-conjugated anti-rabbit IgG secondary Ab (1:5000, CST, USA). The peroxidase-positive bands were detected using an ECL detection kit.

### RT-PCR

WT, TLR2^−/−^, TLR4^−/−^ and TLR2^−/−^/4^−/−^ DKO mouse macrophage cell lines were treated for various times with rLsa21 (2 μg/ml), LPS (500 ng/ml) or Pam3CSK4 (20 ng/ml). After treatment, cell culture supernatants were removed for cytokine analysis and the cells were recovered in 500 μl of TRIzol (Invitrogen, Carlsbad, CA) and equal volumes of chloroform were added; samples were centrifuged at 12000 rpm for 5 min at 4 C. The aqueous phase was then passed through RNA easy mini columns (MN) and RNA was purified following manufacturers protocol. RNA quality was checked by running on a Formaldehyde gel for 16 s and 18 s RNA bands and also on Bioanalyser. The RNA quantity was assessed by UV spectroscopy and purity by 260/280 ratio. First-strand cDNA was synthesized using superscript III RT system (Invitrogen) following manufacturer’s instructions. RT-PCR was performed in 96 well microtiter plates on an ABI 7500 sequence detection system. The two step amplification was performed in a 10 μl reaction volume containing 50ng cDNA, 10 μM each primer and SYBR green (Roche). Samples were run in triplicate and data were analyzed with Sequence Detection System (Applied Biosystems). The experimental data were presented as fold changes of gene expression of stimulated cells at various time points relative to control. mRNA levels of the analyzed genes were normalized to the amount of β-actin or GAPDH present in each sample^58^. All primers were synthesized by IDT and their sequences are given in Table 1 (Supp. Data).

### Statistical Analysis

For all the experiments, wherever required, Student’s t-test and one way ANOVA were executed for the analysis of the results. The data were represented as the mean of triplicates ±  SEM. p < 0.05 was considered as significant.

## Additional Information

**How to cite this article**: Faisal, S. M. *et al*. *Leptospira* surface adhesin (Lsa21) induces Toll like receptor 2 and 4 mediated inflammatory responses in macrophages. *Sci. Rep.* **6**, 39530; doi: 10.1038/srep39530 (2016).

**Publisher's note:** Springer Nature remains neutral with regard to jurisdictional claims in published maps and institutional affiliations.

## Supplementary Material

Supplementary Data
