# Supplementary material for: Leptospira surface adhesin (Lsa21) induces Toll like receptor 2 and 4 mediated inflammatory responses in macrophages
Source: Sci Rep. 2016 Dec 20;6:39530. doi: 10.1038/srep39530 (PMC5172228; doi:10.1038/srep39530)
Supplement: Supplementary Data [file srep39530-s1.pdf]

Supplementary Information

Leptospira surface adhesin (Lsa21) induces Toll like receptor 2 and 4 mediated inflammatory responses in macrophages

Syed M. Faisal[1¶*], Vivek P. Varma[1¶], M. Subathra[1¶],, Sarwar Azam[1], [1]Anil K. Sunkara[1], Mohd Akif[2], Mirza. S. Baig[3], Yung-Fu Chang[4],

[1] Zoonotic and Infectious Diseases Lab, National Institute of Animal Biotechnology, Hyderabad, India.
[2]Department of Biochemistry, School of Life Sciences, University of Hyderabad, Hyderabad, India
[3]Centre for Biosciences and Biomedical Engineering, Indian Institute of Technology, Indore, MP, India
[4]Department of Population Medicine and Diagnostic Sciences, College of Veterinary Medicine, Cornell University, Ithaca, NY.

¶ Authors share equal contribution

*Corresponding author: Syed M. Faisal, National Institute of Animal Biotechnology, Hyderabad-500049, India. Tel- 91-4023049423, Email-faisal@niab.org.in

The supplementary file contains Figure S1, Figure S2 and its legend and Table 1.

Figure S1

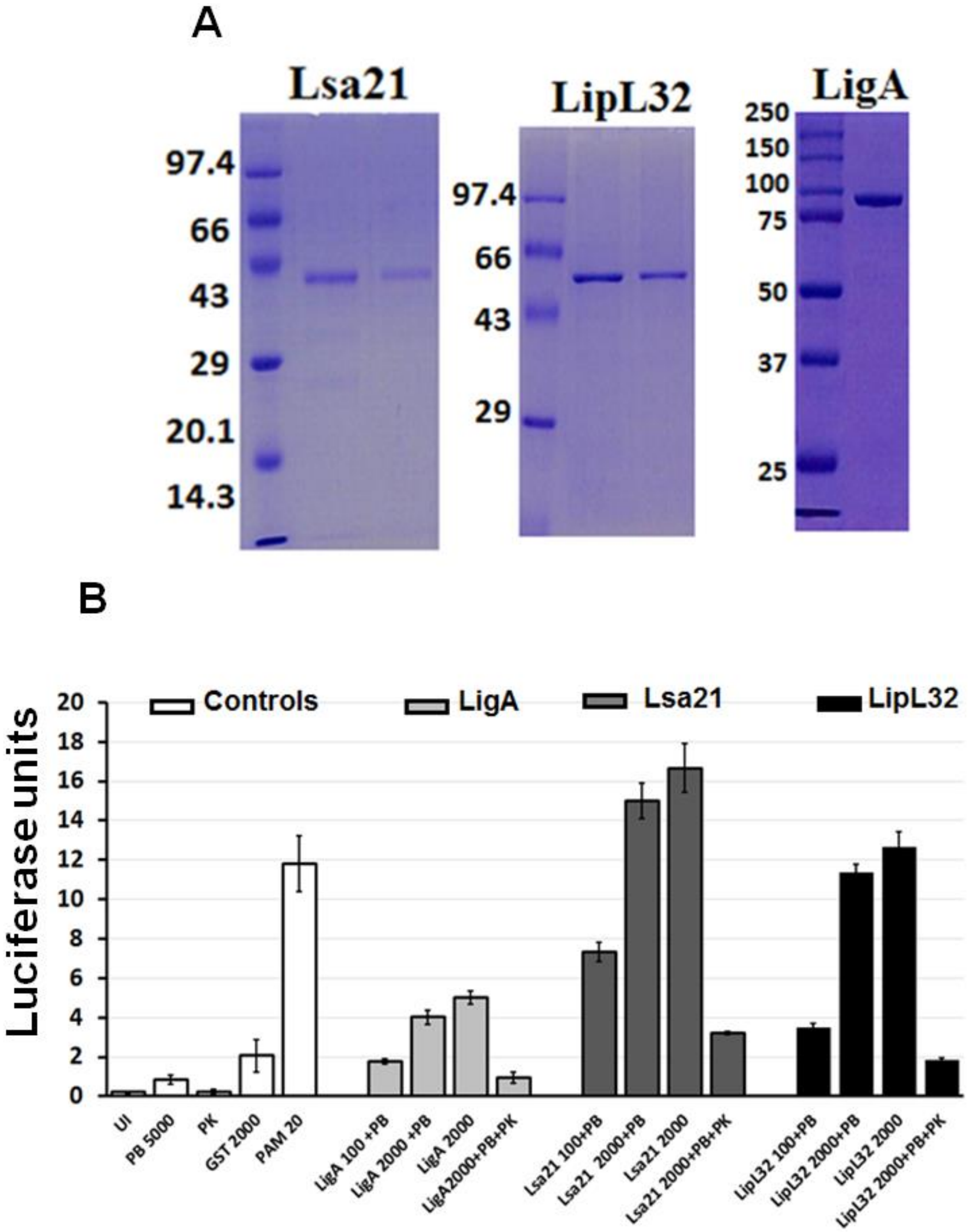

Figure S1. ***Purification and testing of TLR activity of recombinant Leptospira surface proteins.*** (A) GST fusion *Leptospira* surface proteins (Lsa21, LipL32 and LigAvar) were purified using standard procedure. (B) Screening of *Leptospira* proteins for TLR2 activity on HEK293 cell lines**.** TLR2 activity measured by using Luciferase assay after stimulation with the proteins as described in material and methods.

Figure S2

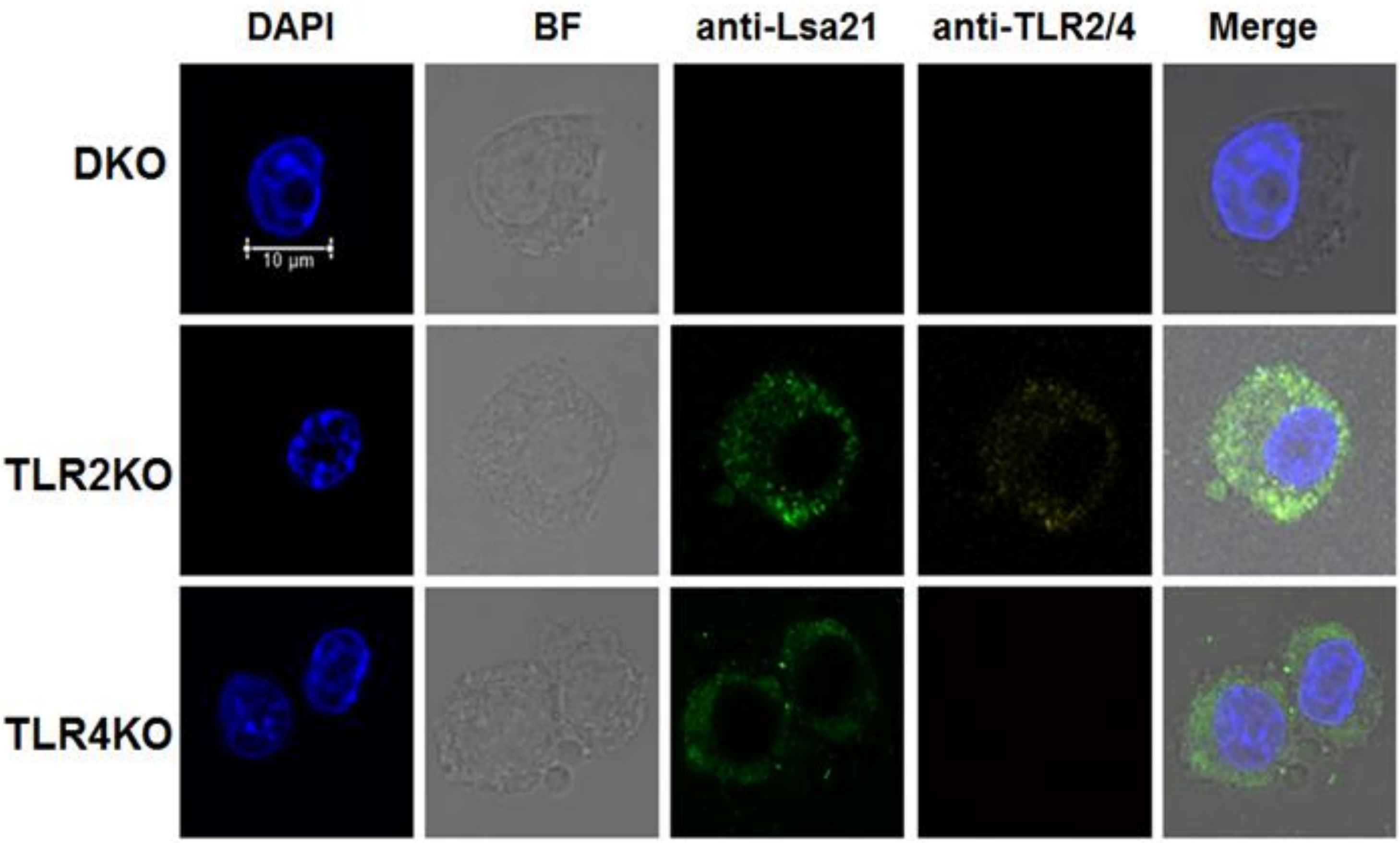

Figure S2. ***rLsa21 interacts with TLR2 and TLR4.*** TLR2$^{-/-}$, TLR4$^{-/-}$ and TLR2$^{-/-}$/4$^{-/-}$ mouse macrophage cell lines were cultured in cell imaging dishes and then treated with rLsa21 (2 µg/ml) for 30 min. After washing, the cells were fixed with 4% PFA and then sequentially incubated with mouse anti-rLsa21 serum and FITC Goat anti-mouse IgG, Alexa Fluor® 647-rabbit anti-mouse TLR2 and PE-rabbit anti-mouse TLR4. After washing, mounting medium (VECTASHIELD® with DAPI) was added and samples were viewed and photographed with confocal fluorescence microscopy. Data are representative of three independent experiments.

Table1. Primers used for RT-PCR

S.NO	Gene	Primer Sequence
1	Beta actin	F- CACCCACACTGTGCCCATCTACGA<br>R- GGATGCCACAGGATTCCATACCCA
2	IL-1b	F- GCCTTGGGCCTCAAAGGAAAGAATC<br>R- GGAAGACACAGATTCCATGGTGAAG
3	Il6	F- TGGAGTCACAGAAGGAGTGGCTAAG<br>R- TCTGACCACAGTGAGGAATGTCCAC
4	TNF-α	F- ATAGCTCCCAGAAAAGCAAGC<br>R- CACCCCGAAGTTCAGTAGACA
5	CCR5	F - ACACTCAGTATCATTTCTGG<br>R- GGATCAGGCTCAAGATGACC
6	Mcp1	F- GGAAAAATGGATCCACACCTTGC<br>R- TCTCTTCCTCCACCACCATGCAG
7	Tlr2	F- CTCCTGAAGCTGTTGCGTTAC<br>R- GCTCCCTTACAGGCTGAGTTC
8	Tlr4	F- TCGCCTTCTTAGCAGAAACAC<br>R- GCCTTAGCCTCTTCTCCTTC
9	Cox2	F- TCTGGAACATTGTGAACAACATC<br>R- AAGCTCCTTATTTCCCTTCACAC
10	Cxcr4	F- GAAGTGGGGTCTGGAGACTATG<br>R- AGGGGAGTGTGATGACAAAGAG
11	Icam2	F- TGCTGTTCTTATTTGTGACATCTG<br>R- TGTATTGAGGCTAAAAAGGAGAGG
12	CCL2	F- ACGTGTTGGCTCAGCCAGA<br>R- ACTACAGCTTCCTTTGGGACACC
13	iNOS	F- CAGCCCAACAATACAAGATGACCC<br>R- CAGTTCCGAGCGTCAAAGACCTGC
14	IFN-g	F- ACTCAAGTGGCATAGATGTGGAAG<br>R- GACGCTTATGTTGTTGCTGATGG
15	CCR4	F- ATCCTGAAGGACTTCAAGCTCCA<br>R- AGGTCTGTGCAAGATCGTTTCATGG
16	CXCR3	F- GCCGGAGCACCAGCCAAGCCAT<br>R- AGGTGGAGCAGGAAGGTGTC
17	CXCL10	F- CATGGTCCTGAGACAAAAGT<br>R- TGATGACACAAGTTCTTCCA
18	STAT 1	F- AGGGGCCATCACATTCACAT<br>R- AGATACTTCAGGGGATTCTC
19	CCL3	F- ACTGCCTGCTGCTTCTCCTACA<br>R- AGGAAAATGACACCTGGCTGG
